# Supplementary material for: The Energy Transfer Yield between Carotenoids and Chlorophylls in Peridinin Chlorophyll a Protein Is Robust against Mutations
Source: Int J Mol Sci. 2022 May 3;23(9):5067. doi: 10.3390/ijms23095067 (PMC9099807; doi:10.3390/ijms23095067)
Supplement: Supplementary file 1 [file ijms-23-05067-s001.zip › ijms-1692863-SI.pdf]

The Energy Transfer Yield Between Carotenoids and Chlorophylls in PCP is Robust Against Mutations

Francesco Tumbarello[1], Giampaolo Marcolin[1], Elisa Fresch[1], Eckhard Hofmann[2], Donatella Carbonera[1], Elisabetta Collini[1*]

[1] Department of Chemical Sciences, University of Padova, via F. Marzolo 1, 35131 Padova, Italy.

[2] Faculty of Biology and Biotechnology, Ruhr-University Bochum, D-44780 Bochum, Germany.

*elisabetta.collini@unipd.it

Supporting Info

Contents:

S1. FROG

S2. Relevant Feynman Diagrams

S3. Fitting Procedures

S3.1. Parallel Model

S3.2 Kinetic Model

S1. FROG

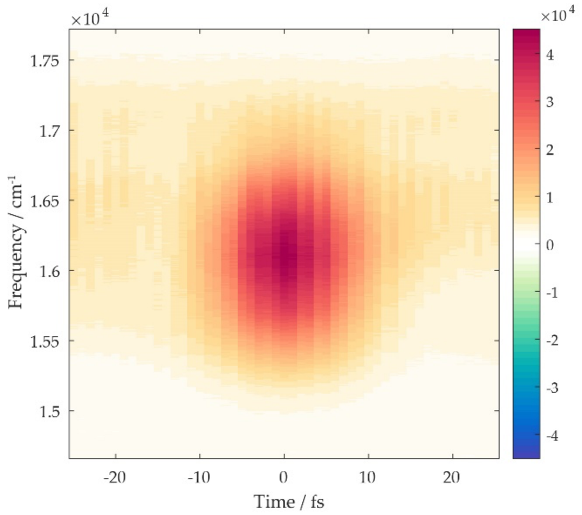

Figure S1. FROG measurement performed at the sample position.

S2. Relevant Feynman Diagrams

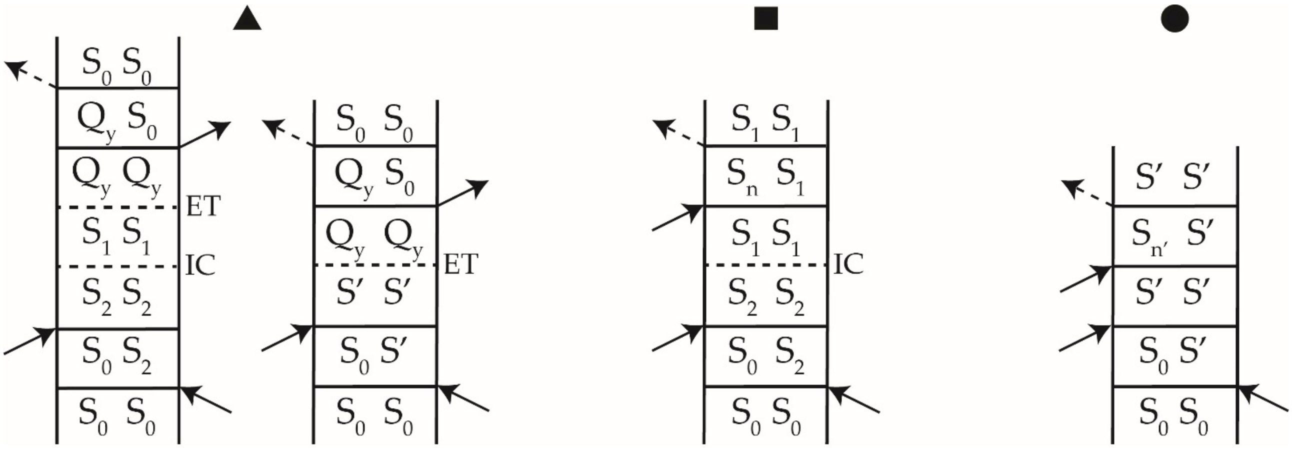

Figure S2. Double-sided Feynman diagrams contributing to the signals discussed in the main text: energy transfer (ET) from Per to Chl a (indicated by a triangle), higher ESA (square) and lower ESA (circle). The time evolution of the density matrix element ab is represented by the two vertical lines, with the time running from the bottom to the top. Solid arrows indicate the interactions with the three laser pulses, while the dashed arrow indicates the emission of the signal. Phenomena occurring during the time t_2 between the second and the third pulse, like internal conversions (IC) and ETs are represented by dashed horizontal lines.

S3. Fitting Procedures

S3.1. Parallel Model

The curves reported in Figures 3b-d of the main text were obtained by fitting the experimental decays with a (multi)exponential parallel model. Each fitting was performed using the following procedures: (i) datapoints collected for $t_2 < 15$ fs were excluded from the fitting to avoid artifacts caused by the pulses overlap; (ii) in order to compensate for possible punctual aberrations of the 2DES maps, all the fitted traces were generated by integrating the signals along the two frequency axes over intervals of 300 cm^{-1} around the central coordinates of the signals; (iii) to prevent the intense beating behavior of the signal from altering the

population fitting results, three damped cosines, representing the three main vibrations of Per[1–3], where included in the fitting function:

$$F(t) = \sum_{i} A_i e^{-t/\tau_i} + \sum_{j=1}^{3} A_j \cos\left(2\pi c \bar{\nu}_j t + \varphi_j\right) e^{-t/\tau_j} \tag{S1}$$

The index *i* runs over the population components: the amplitudes A_i and the time constants τ_i retrieved by the fitting procedures can be found in Table 1. The index *j* refers to the vibrations: the frequencies $\bar{\nu}_j$ and damping times τ_j of the three oscillating components were determined through global analysis[4] of the 2DES signal, while amplitudes A_j and phases φ_j were left as optimizable parameters. The fitted curves in Figures 3b-d show only the population components for clarity.

S3.2. Kinetic Model

The following equations describe the temporal evolution of the populations according to the kinetic model represented in Figure 5a of the main text:

$$S_1(t) = S_{2,CI}(0) e^{-k_{S_1 Q_y} t} \tag{S2}$$

$$S'(t) = -\frac{k_{S',R}}{k_{S',R} - k_{S'Q_y}} S_{2,T}(0) e^{-k_{S',R} t} + \left(S'(0) + \frac{k_{S',R}}{k_{S',R} - k_{S'Q_y}} S_{2,T}(0) \right) e^{-k_{S'Q_y} t} \tag{S3}$$

$$Q_y(t) = \left(S'(0) + \frac{k_{S',R}}{k_{S',R} - k_{S'Q_y}} S_{2,T}(0) \right) \left(1 - e^{-k_{S'Q_y} t}\right) - \frac{k_{S'Q_y}}{k_{S',R} - k_{S'Q_y}} S_{2,T}(0) \left(1 - e^{-k_{S',R} t}\right) + S_{2,CI}(0)\left(1 - e^{-k_{S_1 Q_y} t}\right) \tag{S4}$$

These equations were derived considering that the S_2 state disappears within a time window comparable with the time resolution of the experiment (~ tens of fs). The depletion of S_2 occurs because of the conical intersection (CI), that transfers population to the S_1/ICT state, and the onset of torsional motions, that eventually bring Per to the S′ state. $S_{2,CI}(0)$ and $S_{2,T}(0)$ represent the fractions of S_2 population that reach, respectively, the S_1 state via CI and the S′ state via torsional distortion; $S'(0)$ is the initial population in S′. $k_{S',R}$, $k_{S'Q_y}$ and $k_{S_1 Q_y}$ represent the kinetic constants for the rise of S′, the ET from S′ to Q_y and the ET from S_1 to Q_y, respectively.

The fitting with the kinetic model was performed using the same procedures (i) - (iii) described before for the parallel model. The population dynamics of the higher (detection frequency = 16700 cm^{-1}) and the lower (detection frequency = 15850 cm^{-1}) ESA signals, attributed to the ESA from S_1 and the ESA from S′, were fitted using the equations:

$$ESA(S_1)(t) = -c_{S_1} S_1(t) = -I_{S_{2,CI}}(0) e^{-k_{S_1 Q_y} t} \tag{S5}$$

$$ESA(S')(t) = -c_{S'} S'(t) = \frac{k_{S',R}}{k_{S',R} - k_{S'Q_y}} I_{S_{2,T}}(0) e^{-k_{S',R} t} - \left(I_{S'}(0) + \frac{k_{S',R}}{k_{S',R} - k_{S'Q_y}} I_{S_{2,T}}(0) \right) e^{-k_{S'Q_y} t} \tag{S6}$$

where c_{S_1} and $c_{S'}$ are constants representing the proportionality between the populations in S_1 and S′ and the intensity of the ESA signals from these two states; they account for the dipole moments of the transitions involved in the generation of the signal and for laser intensities at the frequencies corresponding to these

transitions. The parameters $I_{S_{2,T}}(0)$, $I_{S'}(0)$ and $I_{S_{2,CI}}(0)$ optimized in the fitting procedures thus represent the initial intensities $I_{S_{2,T}}(0) = c_{S'}S_{2,T}(0)$, $I_{S'}(0) = c_{S'}S'(0)$ and $I_{S_{2,CI}}(0) = c_{S_1}S_{2,CI}(0)$. **Tab. S1** summarizes the results of the fitting procedures for the two ESA signals.

Table S1. Parameters of the kinetic model described in the main text and in S3.2. They are determined by fitting the temporal traces at (17066,16700) cm^{-1} (higher ESA) and (17066,16850) cm^{-1} (lower ESA).

	**WT**	**N89L**
$\boldsymbol{I_{S_{2,T}}(0)}$	0.57	0.74
$\boldsymbol{I_{S'}(0)}$	0.66	0.63
$\boldsymbol{I_{S_{2,CI}}(0)}$	0.67	0.60
$\boldsymbol{k^{-1}_{S',R}}$/ fs	409	395
$\boldsymbol{k^{-1}_{S'Q_y}}$/ fs	501	503
$\boldsymbol{k^{-1}_{S_1Q_y}}$/ ps	~3	~8

Once the relevant parameters of the kinetic model were determined by fitting of the two ESA signals, they were used to fit the signal corresponding to the Per-to-Chl *a* ET, at (17066,15100) cm^{-1}, according to the equation:

$$ET(t) = c_{ET}\left(I_{S_{2,CI}}(0)\left(1 - e^{-k_{S_1Q_y}t}\right) + \left(I_{S'}(0) + \frac{k_{S',R}}{k_{S',R} - k_{S'Q_y}} I_{S_{2,T}}(0)\right)\left(1 - e^{-k_{S'Q_y}t}\right) - \frac{k_{S'Q_y}}{k_{S',R} - k_{S'Q_y}} I_{S_{2,T}}(0)\left(1 - e^{-k_{S',R}t}\right)\right) \tag{S7}$$

In this case, the only optimized parameter was the proportionality constant c_{ET}, which accounts for the lower laser intensity at detection frequency of 15100 cm^{-1}. The values retrieved for c_{ET} are 0.22 (WT) and 0.24 (N89L).

References

1. Kish, E.; Mendes Pinto, M.M.; Bovi, D.; Basire, M.; Guidoni, L.; Vuilleumier, R.; Robert, B.; Spezia, R.; Mezzetti, A. Fermi Resonance as a Tool for Probing Peridinin Environment. *J. Phys. Chem. B* **2014**, *118*, 5873–5881, doi:10.1021/jp501667t.
2. Buckup, T.; Motzkus, M. Multidimensional Time-Resolved Spectroscopy of Vibrational Coherence in Biopolyenes. *Annu. Rev. Phys. Chem.* **2014**, *65*, 39–57, doi:10.1146/annurev-physchem-040513-103619.
3. Davis, J.A.; Cannon, E.; Van Dao, L.; Hannaford, P.; Quiney, H.M.; Nugent, K.A. Long-Lived Coherence in Carotenoids. *New J. Phys.* **2010**, *12*, 085015, doi:10.1088/1367-2630/12/8/085015.
4. Volpato, A.; Bolzonello, L.; Meneghin, E.; Collini, E. Global Analysis of Coherence and Population Dynamics in 2D Electronic Spectroscopy. *Opt. Express* **2016**, *24*, 24773–24785, doi:10.1364/oe.24.024773.
